# Communications

**Published:** 1874-01

**Authors:** 


*Communications.*

The editors take pleasure in acknowledging the reception of the following articles, which will appear at an early day.

Astigmatism by S. M. Burnett, M.D,. of Tennessee; some farther thoughts in the uses of Phytolacca Decandra and "Clippings," by C. H. Tidd, M.D., of Ohio; Laryngo Thoracheotomy, by R. J. Preston, M.D., of Virginia; Chloroform and Ether, by J. H. Weir, M.D., of Illinois; Puerperal Mania, by J. W. M. Shattuck, M.D., of Mississippi; Hydrocele, by H. B. Lee, M.D., Atlanta, Ga.

A WEALTHY Russian lady has given $30,000 to the St. Petersburgh Academy of Medicine, to endow a department for the medical instruction of women.

